# Moyamoya Disease: Advances in Diagnosis, Treatment, and Surgical Interventions

**DOI:** 10.7759/cureus.59826

**Published:** 2024-05-07

**Authors:** Pratik S Navandhar, Pankaj Gharde, Raju K Shinde, Tushar Nagtode

**Affiliations:** 1 General Surgery, Jawaharlal Nehru Medical College, Datta Meghe Institute of Higher Education and Research, Wardha, IND

**Keywords:** moyamoya disease, neurosurgical procedures, cerebral revascularization, intracranial arterial diseases, cerebrovascular disorders

## Abstract

Moyamoya disease (MMD) is a rare cerebrovascular disorder characterized by progressive narrowing of the brain's arteries, leading to an increased risk of stroke. The primary susceptibility gene, RNF213, has been identified in individuals of East Asian descent, contributing to ongoing research into potential therapeutic targets. The distinction between idiopathic MMD and secondary forms, such as Moyamoya syndrome (MMS), is discussed, focusing on associated conditions and risk factors. Surgical revascularization emerges as the mainstay of treatment, with direct, indirect, and combination bypass procedures explored. The review delves into advancements in imaging technology for diagnosis and treatment planning, emphasizing non-invasive magnetic resonance examination's role in identifying asymptomatic patients. Additionally, insights into anesthetic care and therapeutic approaches underscore the evolving understanding of this complex disease. The presented information aims to contribute to the ongoing dialogue surrounding MMD, providing a valuable resource for clinicians and researchers.

## Introduction and background

A rare cerebrovascular disorder called Moyamoya disease (MMD) causes the arteries in the brain to narrow, increasing the risk of stroke gradually [1]. The internal carotid arteries within the brain and its branches gradually constrict as a result of this vascular sickness, which can cause blockages and possible consequences, including stroke with the ischemic attack, hemorrhaging stroke, and seizure [2,3]. The radiographic characteristics of MMD include increasing stenosis of the internal carotid artery terminal segment and compensating capillary collaterals [2]. A stroke occurs seldom from this cause [1]. Any slowly progressing arteriopathy that affects the major intracranial arteries may produce a similar collateral network of arteries.

Research into the etiology of the disease and potential therapeutic targets has been facilitated by the discovery that RNF213, in individuals from East Asia, the primary susceptibility gene for MMD, is a gene that encodes an unusual E3 ubiquitin ligase [1]. Japan is where the concept of MMD was first created [4]. Progressive stenosis or occlusion of the internal carotid artery's intracranial segment and its proximal branches is the hallmark of MMD [5,6]. The significant conditions and risk factors linked to MMS include cerebral vasculitis and intracranial atherosclerosis [2,7]. The primary considerations for treating MMD include cerebral blood flow restoration, neurological rehabilitation, and neurological protection. The comprehensive approach includes surgical revascularization, cognitive rehabilitation, and appropriate management through medication [8,9]. The initial line of treatment is revascularization surgery, which is to be practiced after diagnosis as soon as possible to prevent additional cerebral infarction [6,10]. The aim of this study is to comprehensively review existing literature on MMD, with a focus on advancing our understanding of its pathophysiology, identifying genetic and radiographic characteristics associated with the condition, differentiating it from similar disorders, evaluating current treatment strategies, and emphasizing the significance of early intervention in enhancing patient outcomes.

## Review

Search methodology

**Figure 1:**
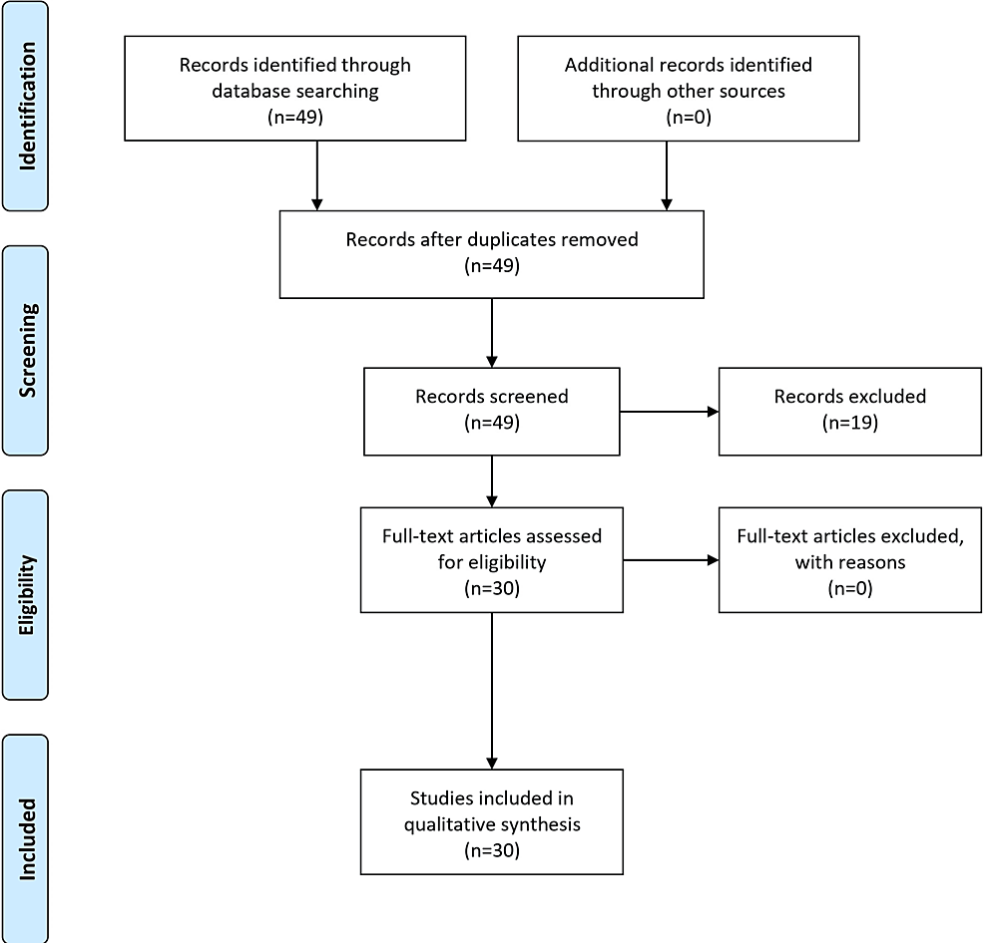
PRISMA flow diagram PRISMA: Preferred Reporting Items for Systematic Reviews and Meta-Analyses

The methodology employed for the literature search involved a comprehensive analysis of scientific literature using pertinent medical publications and databases like Google Scholar and PubMed. Key phrases such as moyamoya disease, moyamoya syndrome, cerebrovascular disease, surgical revascularization, diagnosis, anesthetic care, imaging technology, and therapeutic insights guided the search from 1988 to 2023, covering historical perspectives and contemporary advancements. The inclusion criteria encompassed studies shedding light on MMD diagnosis, treatment, and developments, focusing on surgical treatments, imaging technology, and anesthetic concerns. Exclusion criteria involved filtering out irrelevant articles published outside the specified timeframe, certain study types like editorials or conference abstracts, and articles lacking methodological rigor or presenting duplicate findings. The articles that were not in English were excluded. The PRISMA flow diagram is presented in Figure 1.

MMD is a chronic and progressive disorder of the brain's arteries. These blood vessels narrow in people with MMD, which can eventually induce blockages, ischemic stroke, hemorrhagic stroke, and seizures. Nishimoto's disease was the term given to MMD in Europe following the presentation of the first countrywide data reported by Nishimoto et al. at the Symposium Neuroradiologicum in Paris in 1967 [11]. The term "moyamoya disease" has gained acceptance in the international community since Suzuki et al. published it in the English literature in 1969 [3]. A network of deep thalamus perforating and lenticulostriate collaterals forms to avoid the blockage, giving rise to the angiographic "puff of smoke" appearance [2]. MMD poses a lifetime risk of stroke and neurological death, with a five- to ten-year-old peak age of presentation in children [12]. The racialized clinical and imaging phenotype of MMD is well-defined, and it is a very prevalent cause of juvenile stroke. Patients with MMD do not have a coexisting ailment. Still, those with MMS have Moyamoya in addition to an acquired or genetic disorder, such as trisomy 21, sickle cell disease, or neurofibromatosis type 1 [10]. Patients with Moyamoya illness may be more vulnerable to gestational age-related stroke during pregnancy. Intracranial bleeding is more likely to happen in the antepartum phase, particularly at 24 weeks or less, whereas cerebral infarction is more likely to happen after delivery [13].

Associations and differential diagnoses in MMD

Numerous clinical symptoms, including ischemia deficits, cerebral bleeding, sensory disruption, involuntary movement, seizures, and headaches, are prevalent in MMD patients [1]. Angiographic evidence of bilateral stenosis or occlusion of the terminal portion of the internal carotid arteries, with the formation of basal collateral vessels. Absence of other intracranial vasculopathies that could cause similar angiographic findings. These criteria are essential for confirming a diagnosis of MMD based on the traditional angiographic findings [14]. Children are more likely to experience ischemic symptoms, although cerebral hemorrhage is more common in adults [12]. Children's ischemia episodes are frequently brought on by hyperventilation. The primary cause of intracranial bleeding is hemodynamic stress on the delicate Moyamoya arteries. For individuals with ischemia issues, a variety of surgical techniques have been developed to improve cerebral blood flow, including indirect bypass, direct bypass, and their combination [15]. Thyroid disorders and MMD have been reported to cohabit more frequently in recent years. However, the exact mechanism underlying this coexistence remains unknown [16]. Geographic and ethnic diversity can be seen in MMD and MMS, with a predominance of Moyamoya illness in East Asian cultures [17]. The similar cerebrovascular lesions linked to the following underlying disorders must be ruled out to properly diagnose MMD: atherosclerosis, autoimmune diseases, meningitis, brain tumors, Down syndrome, neurofibromatosis type-1, traumatic brain injury (TBI), and cranial irradiation [14]. The frequency of cardiac symptoms, such as congenital heart abnormalities and coronary artery disease (CAD), was investigated in a predominantly Caucasian group of patients with MMD [18]. MMD features are described in Table 1.

**Table 1:** MMD features Table created by Pratik Navandhar MMD: Moyamoya disease; CAD: Coronary artery disease; TBI: Traumatic brain injury Moyamoya arteries refer to small blood vessels at the base of the brain, which can become blocked or narrowed; indirect bypass is a surgical technique where a new blood supply is created for the brain by rerouting existing blood vessels; direct bypass is a surgical technique where a new blood vessel is directly connected to a cerebral artery to bypass blockages; combination refers to a Surgical technique involving both indirect and direct bypass procedures.

Feature	Description
Clinical symptoms	Ischemia deficits, cerebral bleeding, sensory disruption, involuntary movement, seizures, headaches
Diagnostic criteria	Angiographic findings
Age	Children more likely to experience ischemic symptoms, adults more common to experience cerebral hemorrhage
Ischemia episodes in children	Frequently brought on by hyperventilation
Intracranial bleeding	Caused by hemodynamic stress on Moyamoya arteries
Surgical techniques	Indirect bypass, direct bypass, combination
Co-occurrence with thyroid disorders	Reported, mechanism unknown
Geographic and ethnic diversity	Predominance in East Asian cultures
Differential diagnoses	Atherosclerosis, autoimmune diseases, meningitis, brain tumors, Down syndrome, neurofibromatosis type-1, TBI, cranial irradiation
Cardiac symptoms	Congenital heart abnormalities, CAD

Differential diagnosis for Moyamoya includes conditions such as atherosclerosis, autoimmune diseases, meningitis, brain tumors, Down syndrome, neurofibromatosis type-1, TBI, and cranial irradiation. Several factors are considered to rule out these conditions.

Moyamoya typically affects younger individuals and is not typically associated with the risk factors for atherosclerosis, such as older age, smoking, high cholesterol, and hypertension [1]. Additionally, the blood vessel narrowing pattern in Moyamoya differs from atherosclerosis [13]. Autoimmune diseases can affect blood vessels; they often present with other systemic symptoms and signs specific to the autoimmune condition, which are absent in MMD [3]. Meningitis is an inflammatory condition affecting the meninges, the membranes covering the brain and spinal cord. While meningitis can cause vascular changes in the brain, the underlying causes and symptoms are distinct from those of MMD [17]. Brain tumors can cause symptoms similar to those seen in MMD, such as headaches and neurological deficits [8]. However, imaging studies can typically differentiate between the two conditions based on the characteristic vascular changes in Moyamoya. Down syndrome is a genetic disorder caused by an extra chromosome 21. While individuals with Down syndrome have an increased risk of certain health conditions, including congenital heart defects, they are not predisposed to MMD [5,14]. Neurofibromatosis type-1 is a genetic disorder characterized by the growth of tumors along nerves in the skin, brain, and other body parts [9]. While vascular abnormalities can occur in neurofibromatosis type-1, they differ from those seen in MMD [13,14]. TBI can cause damage to blood vessels in the brain, but the resulting vascular changes and symptoms are typically different from those seen in MMD [11].

Additionally, MMD typically occurs without a history of significant head trauma. Cranial irradiation can damage blood vessels in the brain and may increase the risk of certain vascular conditions. However, the pattern of vascular changes seen in MMD differs from those caused by cranial irradiation [10,14]. Differential diagnosis of MMD is depicted in Table 2.

**Table 2:** Differential diagnosis of MMD Table created by Pratik Navandhar TBI: Traumatic brain injury; MMD: Moyamoya disease

Differential Diagnosis	Ruling Out Factors
Atherosclerosis	Age, smoking, cholesterol, hypertension
Autoimmune diseases	Systemic symptoms specific to the condition
Meningitis	Inflammation of the meninges, distinct symptoms
Brain tumors	Headaches, neurological deficits, differentiated by imaging
Down syndrome	Increased risk of congenital heart defects, not Moyamoya
Neurofibromatosis type-1	Vascular abnormalities differ from Moyamoya
TBI	Different vascular changes and symptoms, no history of trauma in Moyamoya
Cranial irradiation	Vascular changes differ from Moyamoya

In summary, while these conditions may share some symptoms or vascular changes with MMD, carefully considering the patient's clinical presentation, medical history, and imaging findings can help differentiate MMD from other potential causes [12,14]. Imaging studies such as angiography are often crucial in accurately diagnosing MMD.

Surgical revascularization in MMD: approaches, challenges, and outcomes

One of the rare causes of stroke, which that can be effectively treated with surgical revascularization, is MMD. Revascularization can be accomplished surgically by various approaches, each with varying results. These approaches include direct, indirect, and combination bypass procedures [6]. However, due to the heterogeneity of the diseases, different clinical courses, geographic factors associated with the disease, and availability of a wide variety of surgical revascularization procedures, particularly in the pediatric population, selecting the best surgical candidate and technique becomes challenging [19]. If a prompt diagnosis is made and the patient is deemed eligible, cerebral revascularization is an effective treatment for MMD [20]. With the help of this type of therapy, it is possible to stop ischemic episodes or hemorrhages from recurring, which typically result in significant limits on the person's ability to grow personally. Migraine is the most prevalent type of headache; cases of cluster headache, hemiplegic migraine, and tension-type headache have all been documented in MMD [21]. Following surgical revascularization, the majority of patients report relief in their headaches. Nevertheless, some report deterioration or emergence of new headaches following surgery. The gold standard for confirming the diagnosis is cerebral angiography. Treatment modalities are possible, including endoscopic and surgical procedures and conservative management [22]. Surgical revascularization in MMD is described in Table 3.

**Table 3:** Surgical revascularization in MMD Table created by Pratik Navandhar MMD: Moyamoya disease Direct bypass is a surgical revascularization technique where a direct connection is made between a donor artery and a recipient artery in the brain; indirect bypass is a surgical revascularization technique where new blood vessels are encouraged to grow by placing tissue or materials near the brain's surface; combination bypass refers to a surgical revascularization technique that combines aspects of direct and indirect bypass procedures; cerebral angiography is a diagnostic imaging technique that uses X-rays to visualize the blood vessels in the brain; conservative management consists of non-surgical treatment approaches aimed at symptom management and slowing disease progression.

Feature	Description
Treatable cause of stroke	MMD is a rare stroke cause amenable to surgical revascularization.
Surgical revascularization	Various surgical approaches exist for revascularization (direct, indirect, and combination bypass), each with different outcomes
Challenges in selecting treatment	Disease heterogeneity, clinical course variations, geographic factors, and surgical procedure options (especially in children) make selecting the best candidate and technique difficult.
Effectiveness of revascularization	When diagnosed promptly and deemed suitable, cerebral revascularization effectively treats MMD.
Benefits of revascularization	This therapy can prevent recurring ischemic episodes or hemorrhages, which significantly impact a person's growth potential.
Headaches in MMD	Migraine is the most common headache type, with cases of cluster headache, hemiplegic migraine, and tension-type headache also reported in MMD patients.
Headache relief after surgery	Most patients experience headache relief after surgical revascularization, but some might experience worsening or new headaches post-surgery.
Diagnosis confirmation	Cerebral angiography is the gold standard for confirming MMD diagnosis.
Treatment options	Treatment modalities include endoscopic and surgical procedures, along with conservative management.

Advancements in imaging technology for diagnosis and treatment planning in MMD

The ability to identify asymptomatic MMD patients who have not undergone cerebrovascular episodes has been enhanced with the invention of a non-invasive magnetic resonance examination [23]. Due to the small sample size and brief follow-up periods, their clinical characteristics, prognosis, and course of treatment remain unknown. The rare conditions of adult MMD and MMS have a high morbidity and fatality rate [22,24]. MMD is associated with immunological and genetic factors [25]. Neurosurgeons use neuroimaging extensively while treating MMD to minimize postsurgical neurological impairments in patients and optimize surgical success rates during intraoperative navigation and preoperative planning [26]. The clinical use of imaging techniques in the morphological and hemodynamic evaluation of surgical revascularization in patients with MMD patients, utilizing the most recent imaging technologies, helps surgeons choose bypass arteries, monitor postoperative cerebral perfusion, and evaluate the status of Moyamoya vessels [26]. Let's explore the various imaging modalities used, their advantages and disadvantages, success rates in diagnosis, and associated difficulties as follows:

Digital Subtraction Angiography 

Digital subtraction angiography (DSA) offers high-resolution images of blood vessels, allowing for precise visualization of the narrowed arteries characteristic of MMD. It provides dynamic information about blood flow and collateral circulation [23]. The invasive nature of specific medical procedures poses associated risks, such as stroke, allergic reactions to contrast agents, and radiation exposure. Additionally, DSA is expensive and requires specialized equipment and expertise [23]. DSA is considered the gold standard for diagnosing MMD, with a high success rate in identifying characteristic arterial stenosis and collateral vessel formation [5,22]. Accessibility to DSA facilities may be limited, and the invasive nature of the procedure may deter some patients [26].

MRI

MRI provides detailed anatomical images without the use of ionizing radiation. It can visualize the brain's structural and functional aspects, aiding diagnosis and treatment planning [21]. Limited resolution for small vessels compared to DSA. MRI may not always capture dynamic changes in blood flow as effectively [24]. MRI, especially with advanced techniques like magnetic resonance angiography (MRA) and perfusion-weighted imaging (PWI), has a high success rate in detecting MMD, particularly in pediatric patients [26,27]. MRI may not be suitable for all patients, such as those with metal implants or claustrophobia. Interpretation of MRI images requires expertise in neuroimaging [13,25].

CT Angiography

CT angiography (CTA) offers fast acquisition of high-resolution images, making it useful for emergencies or patients unable to undergo MRI. It provides a detailed visualization of vascular anatomy [11,22]. Exposure to ionizing radiation and potential contrast-induced nephropathy are concerns. CTA may not offer as much detail in assessing collateral circulation as DSA [23]. CTA effectively diagnoses MMD, particularly in adults, with a success rate comparable to DSA [21]. Like MRI, CTA may not suit all patients, and contrast administration can pose risks for specific individuals [27].

Single-Photon Emission CT

Single-photon emission CT (SPECT) provides functional information about cerebral perfusion, which can be valuable in assessing the hemodynamic significance of arterial stenosis in MMD [26]. Lower spatial resolution compared to other imaging modalities. Limited availability and higher cost may be limiting factors [3,26]. SPECT can contribute to diagnosing and managing MMD, particularly in evaluating cerebral blood flow dynamics [20]. Interpretation of SPECT images requires expertise in nuclear medicine, and it may not be routinely used in all centers [20].

Advancements in imaging technology, such as integrating artificial intelligence for image analysis and improved imaging protocols, continue to enhance the accuracy and efficiency of MMD diagnosis and treatment planning [17,26]. However, challenges persist in ensuring accessibility to advanced imaging modalities, minimizing associated risks, and interpreting complex imaging findings accurately. Close collaboration among multidisciplinary teams comprising neurologists, neurosurgeons, radiologists, and technologists is essential for optimizing imaging strategies and improving patient outcomes in MMD management. Imaging modalities for MMD are presented in Table 4.

**Table 4:** Imaging modalities for MMD Table created by Pratik Navandhar DSA: Digital subtraction angiography; MRA: Magnetic resonance angiography; PWI: Perfusion-weighted imaging; CTA: CT angiography; SPECT: Single-photon emission CT; MMD: Moyamoya disease

Imaging Modality	Advantages	Disadvantages	Success Rate	Difficulties
DSA	High-resolution images, dynamic blood flow information	Invasive, expensive, limited accessibility	High	Invasive procedure, radiation exposure
MRI	Detailed anatomical images, no radiation	Limited resolution for small vessels, may not capture dynamic flow	High (especially with MRA and PWI)	Claustrophobia, metal implants, interpretation expertise needed
CTA	Fast acquisition, detailed vascular anatomy	Radiation exposure, contrast risks, less detail on collateral circulation	High (comparable to DSA)	Contrast risks, may not suit all patients
SPECT	Functional perfusion information	Lower resolution, limited availability, high cost	Contributes to diagnosis and management	Interpretation expertise needed, not routinely used everywhere

Advancements in diagnosis, anesthetic care, and therapeutic insights for MMD

The importance of accurately diagnosing MMD in affected patients was clarified by advancements in catheter angiography techniques and the emergence of CT and MRI technologies [27]. Neuroimaging is crucial in diagnosing various neurological conditions, including MMD. In MMD, characterized by progressive stenosis or occlusion of the arteries at the base of the brain, imaging techniques are indispensable for accurate diagnosis and treatment planning. Here's how imaging contributes to the diagnosis of MMD:

Visualization of Vascular Anatomy and Assessment of Cerebral Blood Flow

Imaging modalities such as MRA, CTA, and DSA provide detailed visualization of the cerebral vasculature [3,21]. These techniques allow clinicians to identify the characteristic narrowing of the internal carotid arteries and the development of collateral vessels, which are hallmarks of MMD [25]. Beyond anatomical changes, MMD often leads to alterations in cerebral blood flow. Perfusion imaging techniques, such as PWI in MRI and perfusion SPECT, enable the assessment of cerebral perfusion patterns [24,26]. This information is vital for evaluating the hemodynamic significance of arterial stenosis and assessing the risk of ischemia or stroke.

Differentiation from Other Conditions and Monitoring Disease Progression

MMD shares clinical features with other cerebrovascular disorders like atherosclerosis or vasculitis. Imaging helps distinguish MMD from these conditions by demonstrating the characteristic pattern of arterial narrowing and collateral vessel formation unique to MMD [26]. Serial imaging studies are valuable for monitoring disease progression over time. Repeat imaging allows clinicians to assess changes in arterial stenosis, collateral circulation, and cerebral perfusion, which can inform treatment decisions and prognosis [7, 9, 23].

Surgical Planning and Post-treatment Evaluation

For patients undergoing surgical intervention, such as revascularization procedures (e.g., bypass surgery), preoperative imaging is essential for surgical planning [19,24]. Detailed anatomical information from imaging helps surgeons identify suitable donor vessels, plan the optimal surgical approach, and anticipate potential intraoperative challenges. Imaging evaluates treatment efficacy and monitors for complications after surgical or medical treatment. Post-treatment imaging allows clinicians to assess changes in vascular anatomy, cerebral perfusion, and the resolution of symptoms [26,27].

Neuroimaging is a cornerstone in diagnosing and managing MMD by providing detailed information about vascular anatomy, cerebral perfusion, and disease progression. It enables clinicians to make accurate diagnoses, develop personalized treatment plans, and monitor patients' response to therapy, ultimately improving outcomes for individuals affected by this rare cerebrovascular condition. As our understanding of MMD has grown over time, so too has the anesthetic care of its patients. Optimal anesthesia care for MMD patients requires meticulous attention to neurological status and cerebral perfusion. Preoperative assessment is crucial to gauge vascular compromise and neurological function [3,27]. Maintaining adequate cerebral perfusion during anesthesia induction and maintenance by avoiding hypotension and hypoxia is paramount. Neuroprotective measures, such as normocapnia and normothermia maintenance, help prevent further brain ischemia [9,10]. Monitoring cerebral perfusion and intracranial pressure is essential to avert ischemia or hemorrhage [21]. Anesthetic agents with minimal impact on cerebral autoregulation and metabolism, like propofol and opioids, should be preferred.

Additionally, considering the patient's comorbidities and medications is vital for tailored anesthesia management. Collaborative efforts among neurologists, neurosurgeons, and anesthesiologists are imperative for optimizing perioperative care and reducing the risk of neurological complications in MMD patients [27,28]. These have arisen particularly from the identification of risk factors for anesthetic agent-related perioperative complications and outcomes [28]. When considering anesthesia in patients with MMD, there are several risk factors and potential complications to be aware of.

Anesthesia effects on Moyamoya patients

Cerebral Perfusion and Hemodynamic Instability

Anesthesia can impact cerebral perfusion pressure and blood flow regulation, which may exacerbate Moyamoya patients' already compromised cerebral circulation [19]. Anesthesia can cause blood pressure and heart rate fluctuations, which may further jeopardize cerebral perfusion in patients with already compromised circulation [11, 22].

Increased Risk of Stroke, Hypercapnia and Hypoxemia, Postoperative Neurological Deficits

Moyamoya patients are at an increased risk of stroke due to their underlying vascular pathology. Anesthesia and surgery can further increase this risk, especially if there are sudden changes in blood pressure or blood flow during the procedure [25,29]. Anesthesia-induced respiratory depression can lead to hypercapnia (increased CO_2_ levels) and hypoxemia (reduced oxygen levels), which may exacerbate cerebral ischemia in Moyamoya patients [27,28]. Moyamoya patients are more susceptible to postoperative neurological deficits, including transient ischemic attacks (TIAs) or strokes, which may occur due to perioperative hemodynamic fluctuations or thromboembolic events [23,26,27]. Currently, no known treatment can stop the disease's progression or even reverse intracranial arteriopathy. Although randomized clinical trials have not been conducted, there are compelling signs that neurosurgical intervention, either direct, indirect, or combination revascularization operations, can lower the risk of ischemic stroke and potentially also improve cerebral perfusion, which could lead to cognitive impairment [30]. The summary of included studies is shown in Table 5.

**Table 5:** Summary table Table created by Pratik Navandhar MMD: Moyamoya disease; MMS: Moyamoya syndrome

Author(s)	Year	Main Characteristics
Ihara et al. [1]	2022	Discusses diagnosis and interventions for MMD, focusing on recent advancements in imaging techniques and surgical approaches
Scott et al. [2]	2009	Reviews MMD and MMS, emphasizing the importance of early diagnosis and multidisciplinary management for optimal patient outcomes
Esin et al. [3]	2016	Provides information on MMD, including epidemiological data, clinical features, and treatment modalities, with a focus on the Russian patient population
Hishikawa et al. [4]	2016	Reviews clinical research on MMD, highlighting recent findings on genetic predisposition, pathophysiology, and novel therapeutic targets
Shang et al. [5]	2020	Discusses progress in MMD research, exploring emerging biomarkers, imaging modalities, and potential pharmacological interventions for disease management
Velo et al. [6]	2022	Reviews Moyamoya vasculopathy, including cause, clinical manifestations, and treatment, with a comprehensive analysis of current surgical and endovascular approaches and their outcomes
Kalashnikova et al. [7]	2023	Discusses MMD and MMS, providing insights into diagnostic challenges, management strategies, and outcomes, particularly in the Russian healthcare context
Zhang et al. [8]	2022	Focuses on progression of MMD, examining the role of angiogenesis, inflammation, and hemodynamic factors in disease progression and potential therapeutic implications
Fujimura et al. [9]	2016	Provides overview of MMD, covering its historical background, epidemiology, pathophysiology, clinical presentation, and treatment options, with an emphasis on recent advancements
Kimiwada et al. [10]	2022	Discusses MMD in infants and toddlers, highlighting unique clinical features, challenges in diagnosis, and optimal treatment strategies tailored to this age group
Tokunaga et al. [11]	2008	Reviews MMD focusing on surgical techniques, perioperative management, and long-term outcomes, with insights from a Japanese clinical perspective
Dlamini et al. [12]	2019	Examines childhood MMD, discussing its clinical spectrum, neuroimaging findings, and optimal management strategies, including the role of revascularization procedures in pediatric patients
Inayama et al. [13]	2019	Studies MMD in pregnancy, elucidating the challenges in diagnosis and management during pregnancy and delivery, with a review of maternal and fetal outcomes and potential complications.
Fujimura et al. [14]	2015	Compares international and regional differences in MMD diagnosis, highlighting variations in epidemiology, clinical presentation, and treatment outcomes across different populations
Farrugia et al. [15]	1997	Reviews MMD, providing a historical perspective, epidemiological data, and insights into the evolution of diagnostic and therapeutic approaches over the years.
Zhang et al. [16]	2023	Reviews research progress on MMD combined with thyroid diseases, exploring the underlying pathophysiological mechanisms, clinical implications, and potential therapeutic targets
Maki et al. [17]	1988	Discusses MMD, presenting a comprehensive overview of its clinical features, neuroimaging findings, and treatment options based on early experiences with the disease
Larson et al. [18]	2021	Studies cardiac manifestations in a Western MMD population, exploring the prevalence, mechanisms, and clinical implications of cardiac abnormalities in patients with MMD
Appireddy et al. [19]	2019	Examines surgery for MMD in children, discussing surgical indications, techniques, perioperative management, and outcomes, with a focus on optimizing neurodevelopmental outcomes in pediatric patients
Peña-Tapia et al. [20]	2006	Discusses MMD, providing a comprehensive overview of its clinical manifestations, diagnostic modalities, and treatment options, with insights from Spanish patient cohorts
Chiang et al. [21]	2022	Reviews diagnosis and treatment of headache associated with MMD, emphasizing the importance of accurate diagnosis, multimodal pain management, and long-term follow-up for headache resolution
Angelozzi et al. [22]	2020	Discusses hemorrhagic MMD, exploring the risk factors, pathophysiological mechanisms, and optimal management strategies for preventing and treating hemorrhagic complications
Kuroda et al. [23]	2015	Reviews asymptomatic MMD and the ongoing AMORE study, discussing the natural history, risk of ischemic events, and rationale behind the ongoing clinical trial investigating optimal management approaches
Gonzalez et al. [24]	2023	Provides current perspectives and future directions for adult MMD and MMS, highlighting emerging treatment modalities, challenges in management, and avenues for future research
Huang et al. [25]	2017	Explores the etiology and pathogenesis of MMD, discussing genetic, environmental, and hemodynamic factors implicated in disease development and progression, with potential implications for targeted therapies
Du et al. [26]	2022	Reviews imaging methods for surgical revascularization in MMD patients, discussing the utility of various imaging modalities for preoperative planning, intraoperative guidance, and postoperative assessment
Scott et al. [27]	2009	Introduces MMD, providing a concise overview of its clinical features, diagnostic modalities, and treatment options, with a focus on raising awareness among clinicians
Parray et al. [28]	2011	Reviews MMD and anesthetic management, discussing perioperative considerations, anesthetic techniques, and potential complications during surgical interventions for MMD
Blanc et al. [29]	2015	Discusses diagnosis, clinical features, evolution, and treatment of MMD in 10 patients, presenting detailed case studies to illustrate the varied clinical presentations and treatment outcomes
Kronenburg et al. [30]	2014	Reviews recent advances in MMD pathophysiology and treatment, summarizing key findings from molecular, genetic, and imaging studies, and discussing implications for future therapeutic interventions

## Conclusions

In conclusion, the increasing constriction of the intracranial internal carotid arteries and their proximal branches characterizes MMD, which poses a distinct and intricate problem within cerebrovascular disorders. The primary treatment, especially for patients with the RNF213 c.14576G>A polymorphism, is surgical revascularization, which includes various bypass techniques. However, as the condition progresses, problems still arise, especially in young children. Advances in surgical techniques, anesthesia, and diagnostic methods provide hope for better outcomes despite ongoing investigations into the pathophysiology and unknown causes of MMD. This review article highlights the significance of an integrated strategy involving neurological surgery, imaging, and anesthetic care to treat MMD comprehensively. Future research should focus on elucidating the underlying mechanisms of MMD, refining surgical interventions, enhancing imaging modalities for early detection, and optimizing perioperative care to improve patient outcomes and quality of life further.
